# Metastasis-associated miR-23a from nasopharyngeal carcinoma-derived exosomes mediates angiogenesis by repressing a novel target gene TSGA10

**DOI:** 10.1038/s41388-018-0183-6

**Published:** 2018-03-09

**Authors:** Lili Bao, Bo You, Si Shi, Ying Shan, Qicheng Zhang, Huijun Yue, Jie Zhang, Wei Zhang, Yunwei Shi, Yifei Liu, Xin Wang, Dong Liu, Yiwen You

**Affiliations:** 1 Department of Otorhinolaryngology Head and Neck Surgery, Affiliated Hospital of Nantong University, Xisi Road 20, Nantong, 226001 China; 2 Co-innovation Center of Neuroregeneration, Jiangsu Key Laboratory of Neuroregeneration, Nantong University, Qixiu Road 19, Nantong, 226001 China; 3 Department of Pathology, Affiliated Hospital of Nantong University, Xisi Road 20, Nantong, 226001 China

## Abstract

Benefiting from more precise imaging and radiotherapy, patients with locoregionally nasopharyngeal carcinoma (NPC) have a significantly higher survival rate. Nonetheless, distant metastasis is still the predominant mode of failure. Advances in cancer research have highlighted that pathological angiogenesis is necessary for tumor metastasis by offering oxygen, nutrients, or cell metastatic conduits. MicroRNAs (miRNAs), a class of small noncoding RNAs, are increasingly implicated in modulation of angiogenesis in physiological and pathological conditions. Currently, we detected that miR-23a was highly enriched in NPC tissues at the metastatic or premetastatic stage, and its levels in NPC were associated with microvessel density. Subsequently, we proved that alteration of miR-23a expression modulated the growth, migration, and tube formation of HUVECs in vitro and affected the blood vessel outgrowth in the zebrafish model. Considering the possibility that extracellular miR-23a was horizontally transferred from CNE2 cells to HUVECs, we analyzed miR-23a encapsulated in exosomes, showing that overexpression of exosomal miR-23a in NPC promoted angiogenesis both in vitro and in vivo. Moreover, we provided evidences that miR-23a regulated angiogenesis by directly targeting testis-specific gene antigen (TSGA10). Taken together, our findings revealed that metastasis-associated miR-23a from NPC-derived exosomes plays an important role in mediating angiogenesis by targeting TSGA10.

## Introduction

Nasopharyngeal carcinoma (NPC), the most common cancer in head and neck regions, represents a serious health problem in Southern China, Northern Africa, and Alaska [1, 2]. As it is a squamous-cell carcinoma, radiotherapy (RT) is the primary treatment for an early stage, achieving a 5-year overall survival of 84–90% [3]. For locoregionally advanced NPC, chemotherapy–radiotherapy (CRT), reducing the mortality by 18% and increasing the 5-year overall survival by 4–6%, is indisputably the standard treatment method [4, 5]. However, NPC has a high metastatic potential, and distant metastasis is the predominant mode of failure [6, 7]. Disappointedly, the molecular mechanisms for NPC metastasis remain obscure.

Angiogenesis is the growth of new blood vessels sprouting from pre-existing ones. It involves sophisticated orchestration of endothelial cells (ECs) activities, including proliferation, migration, invasion, adhesion, and differentiation [8, 9]. Angiogenesis occurs under normal or pathological conditions, and pathological angiogenesis is necessary for tumor metastasis by offering oxygen, nutrients, or cell metastatic conduits [8 10]. It was supposed that studies on tumor angiogenesis advance the understanding of tumor metastasis. In a recent study, Zhuo et al. described that the microvessel density (MVD) in metastatic NPC patients (49.90 ± 7.25) was significantly higher than that in non-metastatic NPC patients (42.86 ± 7.84) (*P* < 0.05), which encourages us to further study angiogenesis in NPC [11].

MicroRNAs (miRNAs) are a class of small noncoding, single-stranded RNAs that silence gene expression by binding to the 3′ untranslated regions (3′UTRs) of target messenger RNAs (mRNAs) [12]. It is estimated that miRNAs regulate the production of more than one-third of human mRNAs, which may be involved in diverse biological processes [13, 14]. Advances in cancer research have highlighted the mediation of miR-23a–27a–24-2 clusters in angiogenesis. They showed that miR-23a–27a–24-2 cluster members were enriched in ECs and highly vascularized tissues, and inhibition of miR-23/27 repressed sprouting angiogenesis by targeting Sprouty2 and Sema6A proteins [15]. A recent study from Ruan et al. described that overexpression of miR-23a attenuated TNF-α-induced EC apoptosis [16]. Given that angiogenesis is a complex and multistep process, it will be interesting to further investigate the function and mechanisms responsible for miR-23a-mediated regulation of angiogenesis.

Exosomes, released by a variety of cells (including cancer cells, mesenchymal cells, fibroblast cells, and immune cells) [17, 18], are extracellular nanovesicles of a size ranging from 50 to 150 nm in diameter [19, 20]. Similar to the cells generating them, exosomes contain functional biomolecules of a cell, including proteins, RNA, and DNA [21–24]. Advanced research gained interest in the miRNA content of exosomes, and they showed that miRNAs loaded in cancer-secreted exosomes can be shuttled to neighboring or distant recipient cells to exert genome-wide regulation of gene expression [25–30].

Intriguingly, Umezu et al. demonstrated that cancer-derived miRNAs could be transported to human umbilical vein endothelial cells (HUVECs) via exosomes to exert biological roles like endogenous miRNA [31]. We previously isolated and identified NPC-specific exosomes in serum, and then added them into the culture medium of HUVECs. The results showed that NPC–exosome accelerated angiogenesis both in vitro and in vivo [32]. Some miRNAs may play a role in regulating the angiogenic process. This study’s goal is to evaluate whether NPC-secreted miR-23a participates in angiogenesis by adapting exosomes.

## Results

### Overexpression of miR-23a in human NPC is associated with metastatic progression in NPC patients

**Fig. 1:**
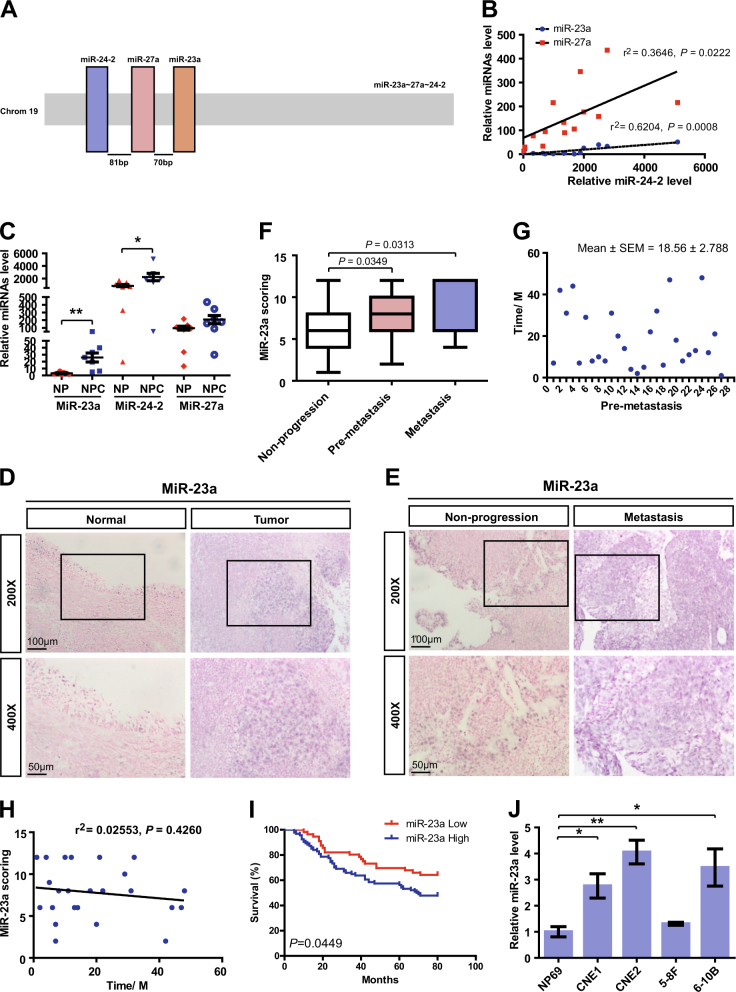
MiR-23a is highly expressed in NPC and associated with metastatic progression. **a** Structure of human miR-23a–27a–24-2 clusters. Chromosome locations of cluster members are shown. The loci of miR-23a, miR-27a, and miR-24-2 are shown as colored boxes. **b** Pearson correlation between miR-24-2 expression and miR-23a/27a expression in nasopharyngeal samples. Linear regression. **c** Levels of miR-23a/27a/24-2 in non-cancerous nasopharyngeal samples and NPC tissues were measured by qRT-PCR. Student’s *t*-test. **d**–**e** Representative images of miR-23a in situ hybridization (ISH) in tissues collected from graph-depicted groups. **f** Statistical comparison of differences in expression of miR-23a in the three groups. Student’s *t*-test. **g** Distribution of time for patients who later developed distant metastasis. **h** Pearson correlation between miR-23a expression and metastasis time (time for patients who later developed distant metastasis). Linear regression. **i** Statistical analyses of the association of miR-23a expression with survival time of the patients. Log-rank test. **j** MiR-23a levels in NP69 and NPC cells were measured by qRT-PCR. Student’s *t*-test

The human miR-23a–27a–24-2 clusters are intergenic on chromosome 19 (Fig. 1a), and Pearson correlation revealed that there was relevant upregulation of miR-23a/27a when miR-24-2 was increased (Fig. 1b). Of note, only miR-23a and miR-24-2 level were significantly altered in freshly obtained NPC tissues by quantitative real-time PCR (qRT-PCR), and miR-23a function was subsequently explored for detecting higher fold change (8.24 vs. 2.73) (Fig. 1c).

To investigate the role of miR-23a in NPC progression, in situ hybridization (ISH) was performed to verify miR-23a expression. It was shown that miR-23a expression level was elevated in NPC specimens and was even higher in metastatic specimens (Fig. 1d–f). Intriguingly, among all metastatic specimens, 84.38% (27/32) were collected from patients who later developed distant metastasis during the 18.56 months of mean follow-up (premetastasis) (Fig. 1g). To explore whether short metastasis time in premetastatic patients exhibits higher miR-23a expression, Pearson correlation analysis was performed, showing no significant difference (Fig. 1h). Collectively, our clinical data suggest that miR-23a could serve as a tissue-based marker for predicting NPC metastasis rather than metastasis time. In addition, the association of miR-23a expression and the prognosis of NPC patients was analyzed, showing that the overall survival time of NPC patients was shorter in the groups exhibiting high miR-23a expression (Fig. 1i). The expression levels of miR-23a were further validated by qRT-PCR, showing that miR-23a was enriched in NPC cell lines (Fig. 1j). Importantly, among a panel of NPC cell lines, CNE2 cells were chosen in subsequent experiments for the highest miR-23a level.

### MiR-23a modulates angiogenesis in NPC

**Fig. 2:**
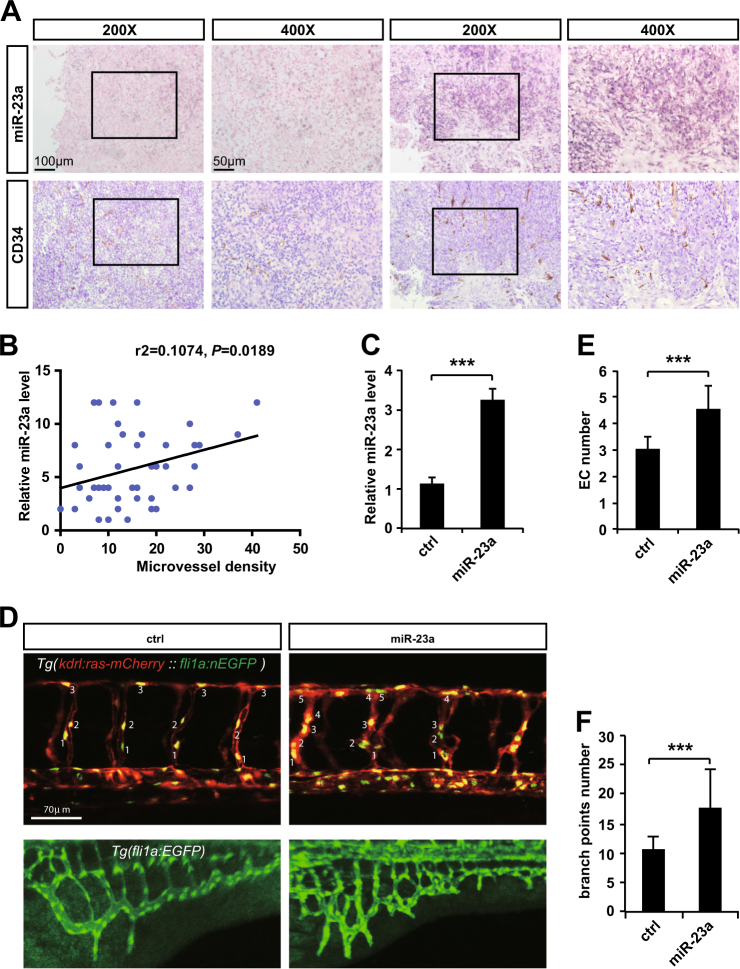
MiR-23a, positively correlated with MVD in NPC, promotes angiogenesis in zebrafish. **a** Representative images of immunohistochemical staining for CD34 with high or low levels of miR-23a. **b** Pearson correlation between miR-23a expression and MVD. Linear regression. **c** Quantitative PCR shows that the precursor enhanced miR-23a efficiently. *T* test. **d** Images of relative embryos injected as indicated. **e** Nuclei of ECs in ISVs are numbered. *T *test. **f** Statistical analysis of branch point number. *T* test

**Fig. 3:**
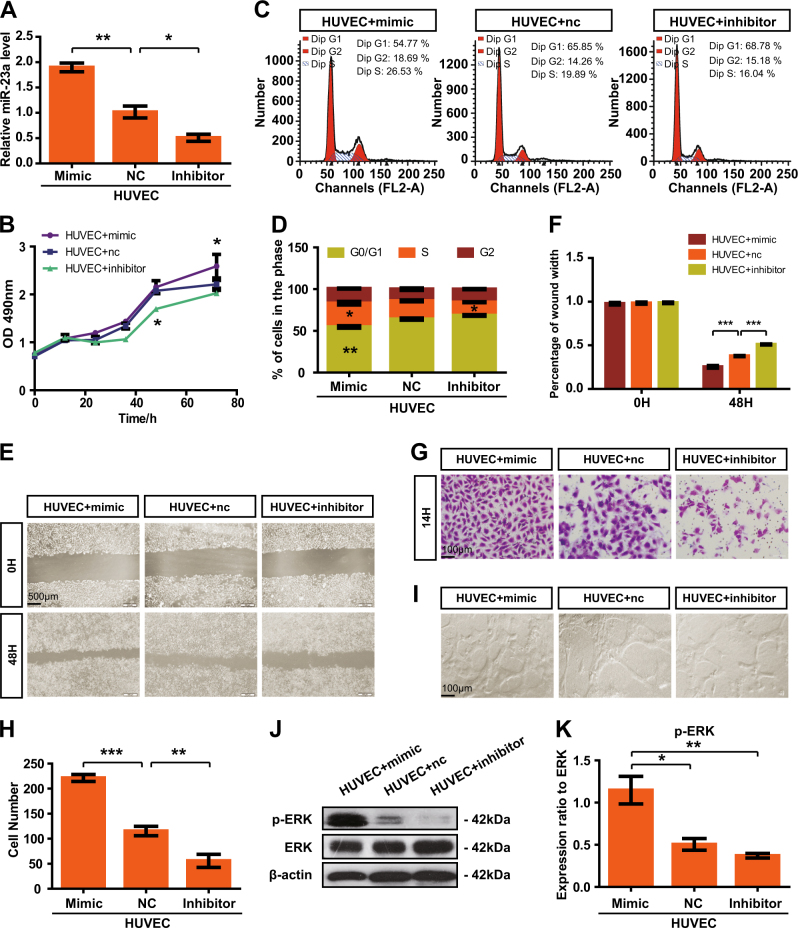
MiR-23a enhances HUVEC proliferation, migration, and tube formation. **a** Transfection efficiency was measured by qRT-PCR. One-way ANOVA. **b** The cell growth of transfected HUVECs was measured by CCK8 assay. Two-way ANOVA. **c**,** d** Flow cytometry analysis of the cell cycle was performed at 36 h after transfection. The graph summarizes the results of three independent experiments. One-way ANOVA. **e**,** f** Wound-healing assay showed cell migration in transfected HUVECs. Two-way ANOVA. **g**,** h** Transwell migration assays were performed to measure cell migration. Cell numbers were calculated as the average of 10 randomly picked fields. One-way ANOVA. **i** HUVECs were inoculated in Matrigel and the indicated images were captured. **j**,** k** Analyses of p-ERK expression in transfected cells

Given that MVD was significantly higher in metastatic NPC patients than that in non-metastatic ones, there might be a potential relationship between miR-23a expression and MVD. In order to verify our hypothesis, ISH and IHC analyses were carried out on the same two independent sets of 51 human NPC specimens, showing a marked correlation between high miR-23a expression score with increased MVD (Fig. 2a, b), which prompted us to further assess the function of miR-23a in angiogenesis. Interestingly, after injecting miR-23a precursor and control miRNA, respectively, into single-cell-stage zebrafish, we demonstrated that miR-23a promoted the outgrowth of subintestinal vessels (SIVs) and EC proliferation in intersegmental vessels (ISVs) of zebrafish (Fig. 2c–f). To further verify the positive effects of miR-23a on angiogenesis, miR-23a mimic, negative control (nc), and an inhibitor were subsequently transfected to obtain HUVECs with different levels of miR-23a (Fig. 3a). Among a series of cellular analyses, we found that upregulation of miR-23a enhanced cell growth, migration, and tube formation of ECs, while silencing miR-23a expression attenuated these functions (Fig. 3b–i). In addition, western blot analysis showed that elevated expression of miR-23a positively regulates ERK signaling (Fig. 3j, k).

### MiR-23a secreted by NPC cells can be transferred to ECs via exosomes

**Fig. 4:**
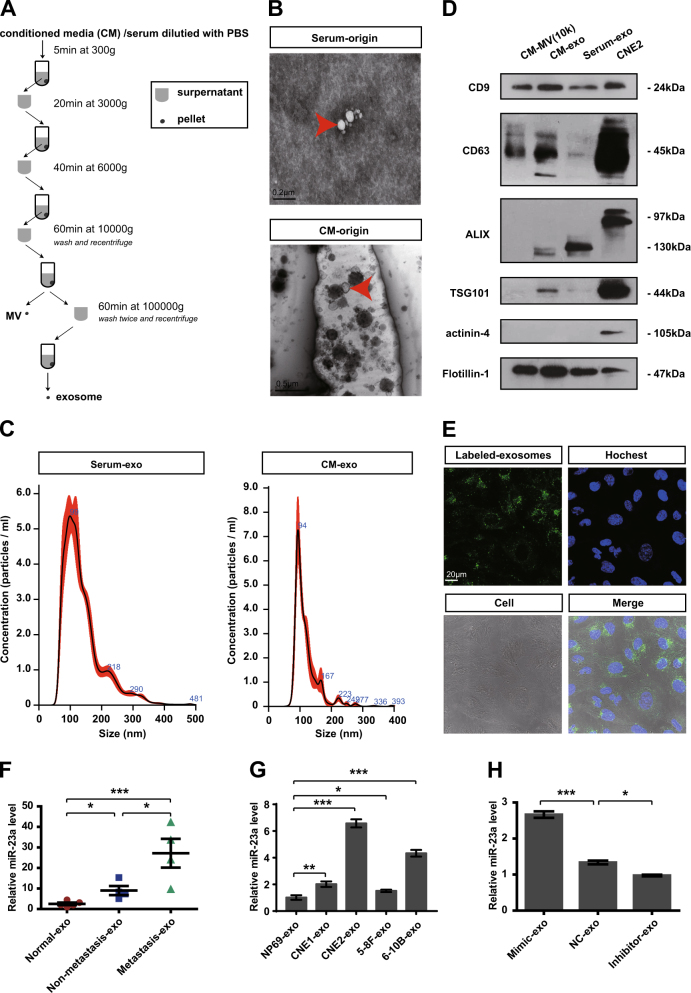
MiR-23a is highly expressed in NPC-exo. **a** Scheme of exosome isolation by differential ultracentrifugation. **b** Representative electron microscopy image of NPC-exo. **c** Nanoparticle tracking analysis displayed the size distribution of exosomes isolated from NPC. **d** Western blot analysis of exosomal markers. CNE2 cell line was used as controls for exosomes characterized. Flotillin-1 was used as a loading control. **e** Uptake of exosomes in HUVECs by confocal microscopy. Blue: Hoechst staining; green: PKH67-labeled exosomes. **f**,** g** qRT-PCR of miRNA level in exosomes isolated from serum (grouping based on the clinical features of patients at the blood-drawing time) or NPC cells. *T *test. **h** qRT-PCR of miRNA expression in exosomes isolated from miR-23a-treated CM. One-way ANOVA

Our previous work gained insight into exosomes, showing that ECs uptook NPC-exo to introduce pathological angiogenesis. To determine whether exosomes mediated the transfer of miR-23a, exosomes were first isolated from serum of NPC patients (serum-exo) or conditioned media (CM) of NPC cells (CM-exo) by differential centrifugation (Fig. 4a). Transmission electron microscopy (TEM) provided evidence that exosomal lipid bilayer membranes were observed (Fig. 4b). NanoSight for exosomes revealed an average of the mode value of 105.5 ± 5.7 nm (serum-origin) and 93.5 ± 2.4 nm (CM-origin) (Fig. 4c). To confirm the identity of isolated exosomes, specific exosome markers such as CD9, CD63, ALIX, and TSG101 [33] were examined. Western blot analysis showed that these markers were highly enriched in isolated exosomes (Fig. 4d). In addition, after they were cocultured with PKH-67-labeled exosomes for 2 h, the recipient cells (HUVECs) exhibited high uptake efficiency, as detected by fluorescence microscopy (Fig. 4e). All the results indicated that we successfully isolated exosomes and exosomes could be taken up by recipient cells. To quantify the relative levels of miR-23a in collected exosomes, we performed qRT-PCR, showing that the levels of circulating exosomal miR-23a were tumor-specifically upregulated, and thus, were higher in NPC patients than healthy volunteers (Fig. 4f). To validate miR-23a level in exosomes, qEV size- exclusion columns, another recognized method for exosome isolation and purification [34], were also applied. qRT-PCR confirmed elevated exosomal miR-23a level in NPC patients by analyzing exosome-enriched fractions (Fig. S1A–C). Analogously, higher relative values of exosomal miR-23a derived from NPC cells than NP69 cells were observed, which was consistent with cellular miR-23a expression (Fig. 4g and Fig. S1D).

### Circulating exosomes containing miR-23a affect angiogenesis

In order to characterize exosomal pathways as a means of miR-23a-mediated angiogenesis, we divided circulating exosomes of NPC patients into two groups: high miR-23a expression group and low miR-23a expression group (Fig. 4f). Notably, miR-23a level was higher in the presence of HUVECs cocultured with a high-expression group, indicating that circulating exosomal miR-23a might transfer to HUVECs (Fig. S2A). Subsequent in vitro models revealed and incubated with a high-expression group enhanced the proliferation, migration, and tube-like structures of HUVECs (Fig. S2B–K). To further evaluate the mediation of exosomes in angiogenesis, circulating exosomes within Matrigel were subcutaneously injected into nude mice, exhibiting a significant difference in vessel formation between high-expression and low-expression groups (Fig. S3A–C). On the basis of these results, we found that exosomes containing miR-23a modulated angiogenesis.

### Exosomal miR-23a regulates angiogenesis both in vitro and in vivo

**Fig. 5:**
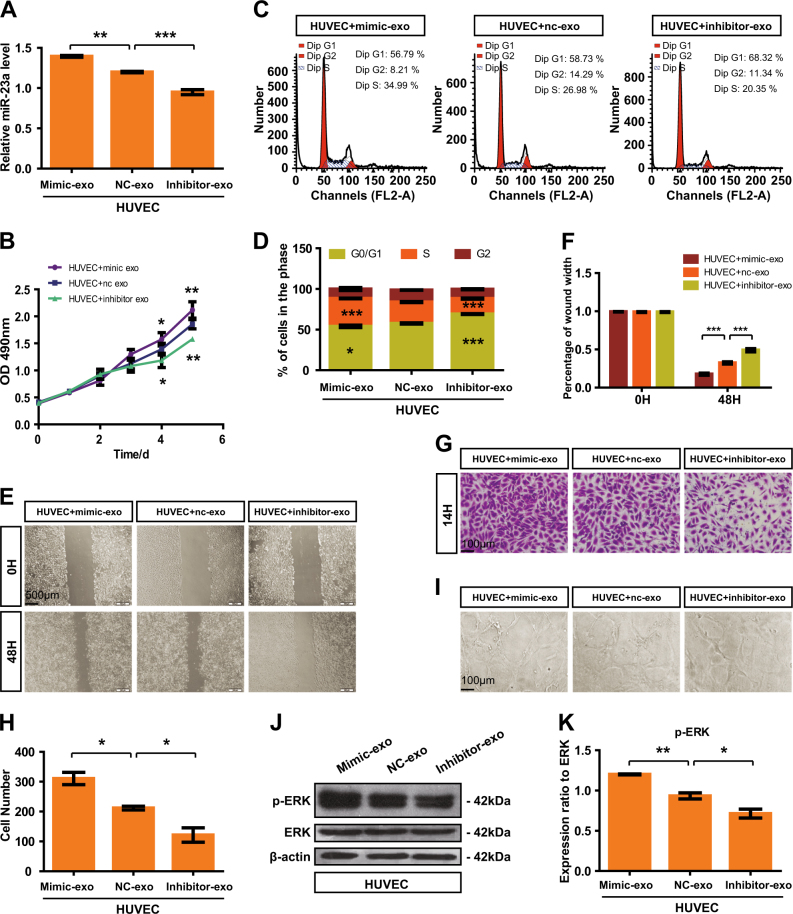
High exosomal miR-23a promoted HUVEC proliferation, migration, and tube formation. **a** Forty-eight hours after treatment with exosomes isolated from CM of transfected CNE-2 cells, miR-23a levels of HUVECs were measured by qRT-PCR. One-way ANOVA. **b** Viabilities of HUVECs treated with various exosomes were measured by the CCK8 assay. Two-way ANOVA. **c**,** d** Cell-cycle analysis was performed 48 h after treatment with exosomes. The graph summarizes the results of three independent experiments. One-way ANOVA. **e**, **f** Wound-healing assay showed the migration of HUVECs treated with various exosomes. Two-way ANOVA. **g**, **h** Transwell migration assays were performed to measure cell migration. One-way ANOVA. **i** Tube formation assays using HUVECs supplemented with exosomes were conducted using Matrigel. **j**, **k** Western blot of p-ERK in HUVEC incubation with exosomes. β-actin was used as a loading control. The results were analyzed with ImageJ software. One-way ANOVA

**Fig. 6:**
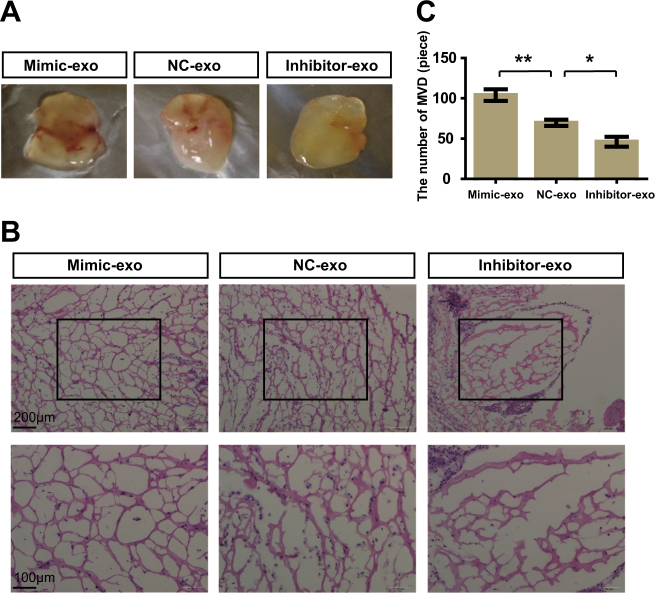
High exosomal miR-23a promoted in vivo angiogenesis. **a** Gross-observation exosomes modulated angiogenesis. **b** Representative micrographs of hematoxylin and eosin staining of Matrigel. **c** Quantitative evaluation of angiogenesis. One-way ANOVA

To exclude the effect due to other angiogenic genes, we attempted to establish a model system to alter exosomal miR-23a expression. First, CNE2 cells with high or low miR-23a expression were obtained after transfection with miR-23a mimic or an inhibitor (Fig. S4A). Of note, CNE2 with higher miR-23a expression enhanced cell proliferation and migration, whereas it failed to influence cell apoptosis (Fig. S4B–L). Then, exosomes in CM were extracted; qRT-PCR confirmed that the exosomal miR-23a expression predominantly changed with respect to transfection (Fig. 4h and Fig. S5). Thus, a relatively high exosomal miR-23a expression was observed for mimic-treated CNE2 cells vs. inhibitor-treated cells. Additional analyses revealed that high exosomal miR-23a markedly accelerated cell viability and migration in CNE2, whereas low exosomal miR-23a attenuated them (Fig. S6), which was similar to the biological role of circulating exosomes (Fig. S7). We then investigated the effects of exosomes isolated from media supplemented with FBS (Gibco) or exosome-free serum (SBI) on angiogenesis. HUVECs cocultured with miR-23a-overexpressing exosomes derived from both media dramatically increased angiogenesis (Figs. 5 and 6 and Fig. S8). These results collectively suggest that exosomes expressing different levels of miR-23a accompanied by angiogenic potential changes; in turn, supports our conjecture that exosomes mediate the transfer of miR-23a.

### MiR-23a directly targets TSGA10 in HUVECs and zebrafish

**Fig. 7:**
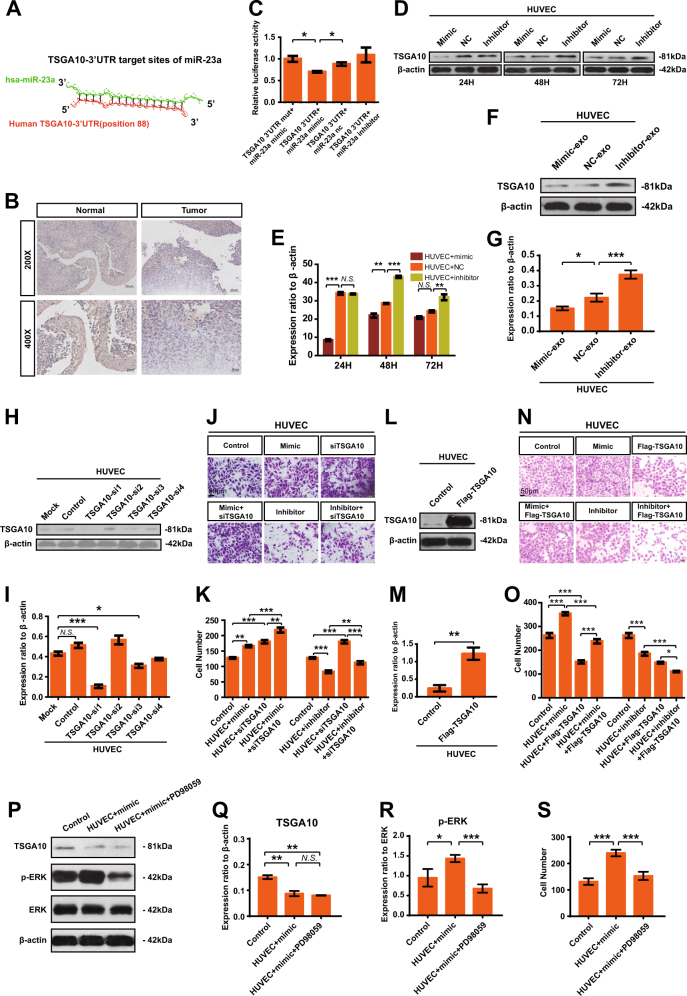
MiR-23a directly targets TSGA10. **a** Schematic of predicted miR-23a binding sequence in *TSGA10*-3′-UTR. **b** Representative IHC images of TSGA10 in tissues collected from graph-depicted groups. TSGA10 staining was mainly localized in the cytoplasm of cells. **c** Overexpression of miR-23a reduced *TSGA10*-3′-UTR luciferase activity in vitro but not mutated *TSGA10*-3′-UTR luciferase activity. Student’s *t*-test. **d**–**g** TSGA10 immunoblotting in HUVECs treated as indicated. The results were analyzed with ImageJ software. One-way ANOVA. **h**, **i** Interference efficiency was detected by western blot. One-way ANOVA. **j**,** k** Transwell migration assays were performed to measure cell migration. One-way ANOVA. **l**,** m** HUVECs treated as indicated and analyzed by western blot. Student’s *t*-test. **n**,** o** Transwell migration assays were performed to measure cell migration. One-way ANOVA. **p**–**r** HUVECs treated as indicated were analyzed by western blot. One-way ANOVA. **s** Transwell migration assays were performed to measure cell migration. One-way ANOVA

MiRNA exerts its biological effect via posttranscriptional gene regulation. As a first step to identify the direct targets of miR-23a, two programs (i.e., MicroCosm Targets Version 5 [35], and RNAhybrid [36]) were applied; and there were hundreds of potential genes predicted. Considering the low specificity of miRNA target prediction, several candidate genes were chosen for possible participation in regulating angiogenesis, including TSAGA10, ARHGAP24, ACVRL1, TIPARP, CCM2, and so on. Among these candidate target genes, testis-specific gene antigen (TSGA10) stood out for the presence of potentially high binding sites (Fig. 7a), relative low expression in NPC samples vs. non-cancerous nasopharyngeal samples (Fig. 7b), and inhibition of angiogenesis [37]. Next, luciferase assays were carried out to test the biologically effective interaction of miR-23a and *TSGA10*-3′-UTR in HUVECs, showing that miR-23a mimic significantly decreased the expression of luciferase*-TSGA10*-3′-UTR (Fig. 7c).

Consistent with the results from the reporter assay, ectopic expression of miR-23a, or treatment with miR-23a-mimic-exo, decreased the protein expression of TSGA10 in HUVECs (Fig. 7d–g). Moreover, TSGA10-si1 was selected (Fig. 7h, i) for transwell assays, revealing that the migration blocked by miR-23a inhibition in HUVECs could be restored by siTSGA10 treatment (Fig. 7j, k). Analogously, the migration enhanced by miR-23a upregulation could be abolished by Flag-TSGA10 treatment (Fig. 7l–o), indicating that miR-23a exerted its function by directly targeting TSGA10. In addition, we undertook to determine whether inhibition of ERK signaling would alleviate the proangiogenic ability of miR-23a. We discovered that blocked ERK signaling using PD98059 rescued cell migration significantly in HUVECs, but not downregulation of TSGA10 (Fig. 7p–s). Collectively, we suggest that miR-23a modulates angiogenesis through directly targeting TSGA10.

**Fig. 8:**
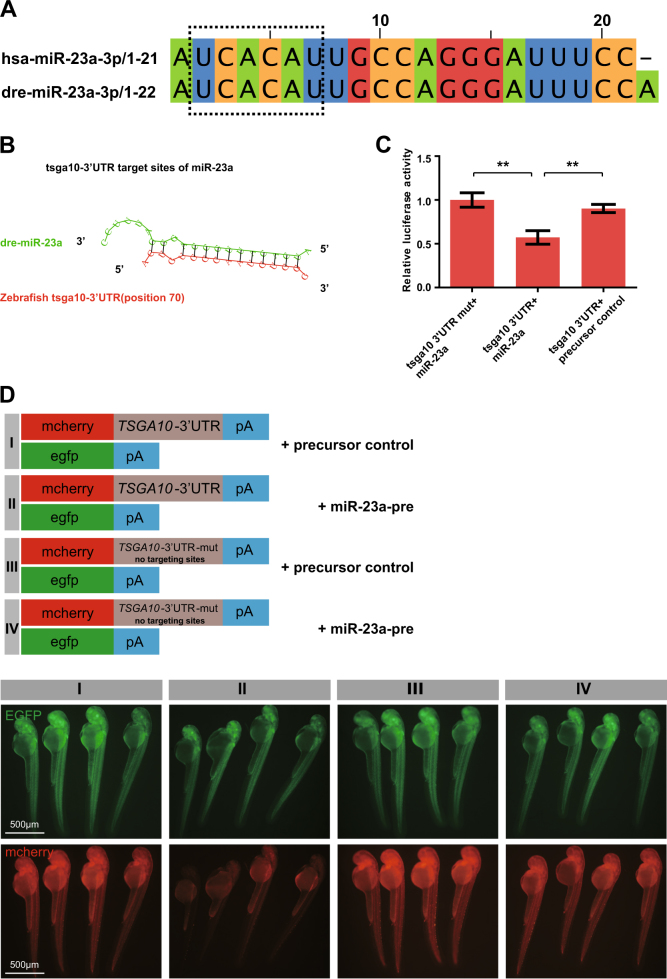
MiR-23a directly targets zebrafish tsga10. **a** The sequences of hsa-miR-23a and dre-miR-23a. Seed sequences are shown in a dashed line box. **b** Schematic of the predicted miR-23a binding sequence in *tsga10*-3′-UTR. **c** Overexpression of miR-23a (miR-23a-pre) reduced *tsga10*-3′-UTR luciferase activity in HUVECs. One-way ANOVA. **d** miR-23a-precursor injection reduced the mCherry levels in mCherry-*tsga10*-3′-UTR sensor, whereas no change in EGFP was observed. In the mutant sensor, mcherry levels were not reduced

Interestingly, in silico analysis also depicted that the 3′-UTR of zebrafish *tsga10* contained the conserved binding sites of miR-23a (Fig. 8a, b). To examine whether miR-23a targeted the 3′-UTR of zebrafish *tsga10* as well, luciferase assays were carried out, showing that miR-23a precursor reduced the luciferase activity of *tsga10*-3′-UTR (Fig. 8c). To further confirm that miR-23a could directly target the zebrafish *tsga10*-3′-UTR, we performed in vivo fluorescence sensor assay, indicating that the overexpression of miR-23a inhibited the *tsga10*-3′-UTR expression (Fig. 8d). These evidences suggest that miR-23a depends directly on zebrafish tsga10 as well.

## Discussion

Distant metastasis is the major reason for treatment failure in NPC [38], and thus, early diagnosis and early treatment for metastasis are critical to improve the outcomes. In this study, we have demonstrated the high expression of miR-23a in metastatic NPC specimens in contrast to non-progression NPC or non-cancerous nasopharyngeal tissues. Moreover, elevated miR-23a expression in premetastatic tissues was found to be a tissue-based marker for the prediction or early diagnosis of NPC metastasis (Fig. 1). Hence, therapies targeting miR-23a, combined with existing conventional antitumor therapies, may serve as an effective treatment approach for NPC patients with high metastatic risk. Understanding the underlying mechanisms responsible for miR-23a-related metastasis may reveal additional strategies for miR-23a intervention.

Metastases require vasculature to obtain oxygen and other nutrients, and thus, blood vessels in tumors are widely accepted as a clinically important therapeutic target [39]. Current antiangiogenic therapy is concentrated largely on blockade of VEGF, and has not yet shown significant survival benefit to patients [40]. Considering that vascular development may depend on various factors at different times [40], we explore the likelihood of miR-23a as a novel antiangiogenic target. On the basis of previous observation that high MVD existed in metastatic NPC patients vs. non-metastatic NPC patients [11], we examined the association of miR-23a and MVD, showing that elevated expression of miR-23a was accompanied by high MVD (Fig. 2). In addition, downregulation of miR-23a in ECs contributed to angiogenesis inhibition by altering cell proliferation, migration, and tube formation (Fig. 3). Our study identifies miR-23a-mediated angiogenesis as one mechanism of miR-23a overexpression associated with NPC metastasis.

It seems to lack a reasonable explanation as to why miR-23a, a proangiogenic factor, is shown to be significantly elevated in NPC cells, rather than blood vessels by ISH. Importantly, accumulating data have demonstrated that exchange of genetic information between cells through various autocrine and paracrine mechanisms is an important approach to intercellular communication and can be transported by exosomes [29, 41]. Compelling evidence indicates that exosomes released from tumor cells have a proangiogenic role associated with the transfer of some characteristic mRNAs, miRNAs, as well as proteins [28, 42–44]. To test the angiogenic activity of miR-23a may be, in part, mediated by exosomes, we quantified the transport of NPC-derived miR-23a to HUVECs, proving that exogenous miR-23a could function like endogenous miR-23a in ECs via exosomal transport (Fig. 4 and Fig. S1). Among a series of in vitro and in vivo analyses, we noted that serum-derived high level of miR-23a transferred to HUVECs via exosomes might further extend their proangiogenic effect to HUVECs with low exosomal miR-23a uptake (Figs. S2–3). Our data offer interests to detect the noncancer source of exosomal miR-23a.

To test whether the increase or reduction of exosomal miR-23a, frequently as a result of regulated expression of miR-23a in CNE2 cells, contributed to angiogenesis, we first modulated miR-23a expression via miR-23a mimic and inhibitor. Of particular interest, miR-23a had been related to tumorigenic activity by facilitating cell growth and migration previously. Hatzl et al. revealed that enforced expression of miR-23a causes reduction of RKIP, thereby inducing a dramatical increase of proliferation in hematopoietic cells [45]. In addition, Yang et al. demonstrated that miR-23a was upregulated and facilitated the progress of cell cycle and epithelial–mesenchymal transition (EMT) in epithelial ovarian cancer cells [46]. To delineate the role of miR-23a within these processes, we carried out cell proliferation assay, wound-healing assay, and transwell assay within transfected CNE2 cells. In agreement with these publications, we showed that upregulated miR-23a induced increased proliferation and motility of NPC cells (Fig. S4). Our study further identifies that miR-23a, a tumorigenic as well as an angiogenic factor, may be a potential treatment target for metastatic NPC patients. In accordance, exosomes of 100,000 × *g*-associated miR-23a were extracted from CM, and cocultured with CNE2, leading to dysregulated cell proliferation and migration (Fig. S6). In analogy, mimic-exo-mediated miR-23a upregulation resulted in the increase of NPC angiogenesis in vitro and in vivo Matrigel plug model (Figs. 5 and 6 and Fig. S8). Our description is the first to establish that exosomal miR-23a mediated NPC angiogenesis and strengthens that exosome-dependent mechanisms may mediate the communication of miR-23a between NPC cells and HUVECs.

Bioinformatics analysis of a region upstream to the miR-23a locus indicated multiple putative binding sites for TSGA10. Since miRNAs exerted their biological function by triggering the degradation of mRNA and/or inhibiting translation [47], we sought to further identify TSGA10 as a direct target of miR-23a. We demonstrated for the first time that miR-23a could directly repress TSGA10 expression through its binding to the specific site in the 3′-UTR of the human TSGA10 gene/zebrafish tsga10 gene. Importantly, compelling evidence indicates that testis-specific gene antigen (TSGA10) inhibits tumor angiogenesis and metastasis [48]. Significantly, in this study, we found that reduced expression of TSGA10 enhanced the migration of ECs, suggesting negatively regulating tumor angiogenesis to miR-23a (Figs. 7 and 8). Our study identifies that proangiogenic functions of miR-23a have been ascribed to direct suppression of the secreted, antiangiogenic factor TSGA10 within ECs, suggesting one mechanism of miR-23a upregulation associated with NPC metastasis.

Overall, our observations suggest that tumor-derived miR-23a may be useful not only to early-diagnosing metastasis, but also to predict future metastasis. We believe that exosomes serve as a paracrine mechanism for miR-23a transported from NPC cells to ECs, thereby accelerating angiogenesis in the adjacent tumor endothelium by directly targeting TSGA10. Our study indicates that a combination of miR-23a inhibitor with currently available VEGF inhibitors would better clarify the disease traits at the individual level; this leads, in turn, to enhance our ability to take preventive treatment for NPC patients with a high risk of metastasis.

## Materials and methods

### Human NPC specimens and ethics statement

The human tissue samples and serum samples were obtained from Affiliated Hospital of Nantong University. The tissue microarrays, containing qualified primary NPC specimens from 150/51 patients, were constructed by OUTDO BIOTECH (Shanghai, China). The inclusion criteria included patients with pathological diagnosis of NPC between June 30, 2006 and October 1, 2008; and patients not receiving any therapy prior to biopsy. The clinical details of 150 patients are shown in Table S1. Our study was carried out with participants who had given their consent previously and the ethical approval of the Institutional Ethics Committee [Approval ID: 2016--101].

### Cell lines and cell culture

Human NPC cell lines (CNE1, CNE2, 5-8F, and 6-10B) and the immortalized normal nasopharynx epithelial NP69 cells were cultured in Otolaryngology Laboratory, Affiliated Hospital of Nantong University. NPC cell lines were maintained in RPMI 1640 containing 10% FBS (Gibco) or 10% exosome-free serum (SBI SystemBiosciences). NP69 cells were maintained in keratinocyte-SFM. CNE2 cell line was recently authenticated by cellular morphology and the short tandem repeat (STR) analysis. HUVECs were maintained in EC medium (ScienCell Research Laboratories).

### Immunohistochemistry and ISH

Immunohistochemistry (IHC) was performed with previously described methods [49] using a 1:500 antibody dilution for CD34 (Abcam). ISH was performed on deparaffinized NPC tissues using LNA™ microRNA ISH miR-23a optimization kit (Exiqon; Woburn, MA) following the manufacturer’s directions. To be specific, on Day 1, we placed the slides in xylene for 15 min. Then, we hydrated them through ethanol solutions to 2× SSC for 1 min each. After incubation in 50 ml of Proteinase K solution at 37 ℃, the slides were washed twice in sterile PBS and dehydrated in ethanol solutions. Place 100 µl of Hyb/probe solution on each slide and immediately place a coverslip with HybridSlip; in turn, incubate the slides at 56 ℃ in a humid chamber dampened with 50% formamide/5× SSC. On Day 2, place the slides in RT 2× SSC on a shaker table for 30 min. After stringent washing in formamide/2× SSC, 2× SSC, RNase buffer, and 1× maleate buffer, respectively, the slides were incubated with anti-DIG antibody in a humid chamber. On Day 3, the slides were incubated with NBT:BCIP and stained with nuclear fast red. Stained slides were assessed according to the staining intensity (strong: 3; moderate: 2; weak: 1; and negative: 0) and the abundance of positive cells (≤5%: 0; 6–25%: 1; 26–50%: 2; 51–75%: 3, and ≥76%: 4) by two pathologists blind to the patient’s clinicopathological information. A final score obtained from the intensity score multiplied by the extent score was used to identify miR-23a expression level. Scores of 0–4 were defined as low expression, and 5–12 were defined as high expression.

### Microvasculature density counting

MVD marked by CD34 was calculated according to the previously described method raised by Wendner et al. [50]. In brief, sections of NPC tissues were observed under light microscopy at low-powered fields (×40 magnification) to identify the high points of MVD. Then, individual microvessels were counted at ×200 magnification in five fields, and the final results represent the mean MVD.

### Western blot analysis

Antibodies against p-ERK, ERK, Flotillin-1, CD63, β-actin (Santa Cruz Biotechnology, CA, USA), TSGA10 (Sigma-Aldrich, America), CD9, ALIX, TSG101, and actinin-4 (Abcam, MA, USA) were used in western blot as previously described [51].

### qRT-PCR

Briefly, cellular RNA and zebrafish RNA were isolated with TRIzol reagent (Invitrogen), while exosomal miRNA was extracted using the Total Exosome RNA Kit (Ambion) and MirVana RNA isolation kit (Ambion) according to recommendation. U6 was used as the corresponding internal reference for qualification of the cellular miRNA. The synthetic spike control (cel-miR-39) served as an invariant control for qualification of the exosomal miRNA.

### Transient transfection with siRNA, plasmid, and miR-23a mimic/nc/inhibitor

Small-interfering RNA (siRNA) for TSGA10 and its negative controls, as well as miR-23a mimic/nc/inhibitor were supplied by Biomics Biotechnologies (Nantong, China). pIRES2-3FLAG-EGFP-TSGA10 (Flag-TSGA10) was supplied by Genechem (Shanghai, China). The sequences used in transfection are

hsa-miR-23a mimic

Sense: AUCACAUUGCCAGGGAUUUCC

Antisense: GGAAAUCCCUGGCAAUGUGAU

hsa-miR-23a nc

Sense: UCACAACCUCCUAGAAAGAGUAGA

Antisense: UCUACUCUUUCUAGGAGGUUGUGA

hsa-miR-23a inhibitor

GGAAAUCCCUGGCAAUGUGAU

TSGA10_siR1

Sense: GCUGGUUGCUAAAGAUCAAdTdT,

Antisense: UUGAUCUUUAGCAACCAGCdTdT;

TSGA10_siR2

Sense: GCGACACCUUGCUAAGAAAdTdT,

Antisense: UUUCUUAGCAAGGUGUCGCdTdT;

TSGA10_siR3

Sense: GCUAAAGCUAAACAAGAAAdTdT,

Antisense: UUUCUUGUUUAGCUUUAGCdTdT;

TSGA10_siR4

Sense: CACAGAACGAGAUAGUCUAdTdT,

Antisense: UAGACUAUCUCGUUCUGUGdTdT.

Twenty-four hours prior to transfection, CNE2 cells or HUVECs were seeded in a six-well plate or a 96-well plate, and siRNAs/plasmids were then transfected using Lipofectamine 2000 (Invitrogen, Carlsbad, CA), while miRNAs were transfected using HiPerFect transfection reagent (Qiagen) at a working concentration following the manufacturer’s directions.

### Wound-healing assay, CCK8, cell-cycle analysis, transwell assay, and apoptosis assay

These experiments were performed as described previously [51, 52]. For apoptosis assay, 1 × 10^6^ cells were harvested, resuspended, and stained with Annexin V-FITC (BD Biosciences, USA) and propidium iodide (Sigma-Aldrich). Flow cytometry was used to analyze apoptotic cells.

### EC tube formation assay

A total of 2 × 10^4^ HUVEC cells pretreated with miR-23a mimic/nc/inhibitor were seeded on BD Matrigel according to the manufacturer’s recommendations. After the cells were attached, supplemented exosomes, or the vehicle control (ECM) was added. Tube-like structures were imaged using an inverted microscope.

### Exosome isolation, labeling, and electron microscopy

Detailed protocols of sequential ultracentrifugation or electron microscopy (EM) can be found in our previous work [52]. For observing the cellular uptake of exosomes, purified exosomes were labeled using PKH-67 labeling kit (Sigma-Aldrich). After coculture with labeled exosomes for 1 h, CNE2 cells/HUVECs were fixed and stained with Hoechst. Pictures were taken with a Leica TCS-SP5 LSM.

For exosomes quantified, a BCA protein assay kit (PIERCE, Rockford, IL, USA) was used to detect protein content. And for *in vitro* experiments, 2 × 10^5^ recipient cells were cocultured with 2 μg of exosomes.

### Isolation of exosomes by size-exclusion chromatography

A commercially available size-exclusion chromatography column (qEV Size Exclusion Columns, Izon Science) was also used for isolation of exosomes following the manufacturer’s directions.

### Nanoparticle tracking analysis

The NanoSight NS300 (Malvern) equipped with sCMOS camera was used for real-time characterization of the vesicles. A 488-nm laser was chosen for each sample, five 60-s videos were recorded and analyzed by NTA software version 3.2, and the final results represent the mean and mode size of vesicles.

### In vivo Matrigel plug assay

A total of 20 μg of purified exosomes mixed with 0.5 mL of BD Matrigel were subcutaneously injected into BALB/c athymic nude mice of 6–8-weeks old. After 7 days, the Matrigel plugs were harvested and stained by hematoxylin and eosin. ImageJ software was used to analyze the vessel area.

### Zebrafish and microinjection and imaging

Zebrafish were obtained from the Zebrafish Center at Nantong University. The study was approved according to Animal Protection Laws of China. The microinjection and imaging were performed as previously described [53].

### Animal ethics statements

All animal experiments carried out in this project were following the NIH Guidelines with the ethical approval of the Administration Committee of Experimental Animals, Jiangsu Province, China [Approval ID: SYXK (SU) 2007-0021].

### Target prediction

Candidate targets of miR-23a were predicted using MicroCosm Targets and RNAhybrid.

### Luciferase assays

Human TSGA10 (ENST00000355053) 3′-UTR (8-197) was inserted into the psiCHECK-2 vector (Promega), in which XhoI and NotI restriction sites were used. The primers used in cloning are

human *TSGA10*-3′-UTR-XhoI-left:

5′-CCGCTCGAGGGCCTAGATCGATCATTAGAAGA-3′;

human *TSGA10*-3′-UTR-NotI-right:

5′-AAGGAAAAAAGCGGCCGCACAGAGATTCAGAGACACAAAGT-3′;

human *TSGA10*-3′-MUTR-XhoI-left:

5′-CCGCTCGAGTGAATCTCTGTTCTAATGTGCCA-3′;

human *TSGA10*-3′-MUTR-NotI-right: 5′-AAGGAAAAAAGCGGCCGCACACTCACTATCACTGCATGGA-3′.

Zebrafish tsga10-3′-UTR and tsga10-3′-MUTR were similarly cloned into the psiCHECK-2 vector.

A Dual-Luciferase Reporter Assay Kit (Promega) was used. Briefly, the psiCheck2*-TSGA10*-3′-UTR or psiCheck2-*TSGA10*-3′-MUTR vector was transfected into HUVECs with miR-23a mimic/nc/inhibitor, or miR-23a-pre/precursor control. Twenty-four hours after transfection, cells were lysed in 20 μl of 1× passive lysis buffer (PLB) (15 min); and then, added to 100 μl of luciferase Assay System Reagent in a white 96-well plate. Reporter activity, normalized to *Renilla* luciferase activity, was measured for 8 s after a delay of 2 s.

### Whole-embryo miRNA sensor assay

The procedure was performed as described previously [53]. Briefly, the pCS2+-*mCherry*-*tsga10*-3′-UTR plasmid was constructed by cloning 3′-UTR of the zebrafish *tsga10* mRNA (ENSDART00000074380) into the pCS2+*-mCherry* vector, whereas the potential target was replaced in mutated pCS2+-*mCherry-tsga10*-3′-UTR. The pCS2+-*egfp* vector was used as an injection control.

### Statistical analysis

Statistical results were calculated with GraphPad Prism^®^ software. Student’s *t*-test, one-way ANOVA, and two-way ANOVA were used to analyze statistical significance, in which *P < 0.05 **P < 0.01, and ***P < 0.001 were considered as significant differences.

## Electronic supplementary material

Supplementary Data
