# Supplementary material for: Metastasis-associated miR-23a from nasopharyngeal carcinoma-derived exosomes mediates angiogenesis by repressing a novel target gene TSGA10
Source: Oncogene. 2018 Mar 9;37(21):2873–89. doi: 10.1038/s41388-018-0183-6 (PMC5966363; doi:10.1038/s41388-018-0183-6)
Supplement: Supplementary file 1 — Supplementary Data [file 41388_2018_183_MOESM1_ESM.pdf]

Supplementary

Table S1. Clinical information of patients in the NPC study, related to Figure 1.

Patient	Stage	Yr. DX.	Age DX.	Gender DX.	Progression (No:1; pre-metastasis:2; metastasis: 3)
1	3	2006.06	18	M	2
2	3	2006.08	67	M	1
3	3	2006.09	48	M	1
4	4	2006.09	59	M	2
5	2	2006.10	44	M	1
6	4	2006.10	71	M	1
7	2	2006.10	57	M	1
8	1	2006.10	52	F	1
9	3	2006.10	30	M	1
10	2	2006.11	45	F	1
11	4	2006.11	48	M	2
12	3	2006.12	65	M	2
13	2	2006.12	34	M	1
14	2	2006.12	33	F	1
15	4	2006.12	25	M	1
16	2	2006.12	45	M	2
17	2	2006.12	38	M	1
18	2	2007.01	39	F	1
19	2	2007.01	57	M	1
20	4	2007.02	41	F	1
21	2	2007.02	75	M	1
22	4	2007.03	50	M	1
23	3	2007.03	30	F	2
24	3	2007.03	46	M	1
25	3	2007.03	49	M	2
26	2	2007.03	43	F	1
27	4	2007.03	38	M	1
28	2	2007.03	46	M	1
29	1	2007.03	32	F	1
30	2	2007.04	71	M	1
31	2	2007.04	70	M	1
32	2	2007.05	34	M	1
33	2	2007.05	39	M	1
34	3	2007.05	51	M	2
35	3	2007.05	72	M	2
36	3	2007.06	39	M	1

37	2	2007.05	46	M	1
38	3	2007.05	53	F	1
39	2	2007.05	39	F	2
40	3	2007.06	38	M	1
41	4	2007.06	50	M	1
42	4	2007.07	45	M	2
43	2	2007.07	40	M	1
44	2	2007.07	43	M	1
45	2	2007.07	65	M	2
46	3	2007.07	38	F	1
47	2	2007.08	38	M	1
48	2	2007.08	51	F	1
49	4	2007.08	62	M	1
50	2	2007.09	51	F	1
51	2	2007.09	69	M	1
52	3	2008.03	44	M	1
53	4	2007.09	52	M	2
54	2	2007.09	35	F	1
55	4	2007.10	46	F	1
56	1	2007.10	33	M	1
57	2	2007.10	52	F	1
58	2	2007.10	52	M	2
59	3	2007.10	42	M	1
60	4	2007.10	41	M	1
61	2	2007.11	53	M	2
62	2	2007.11	35	F	1
63	4	2007.11	51	M	1
64	3	2007.12	52	F	1
65	4	2007.11	50	M	1
66	2	2007.11	44	F	1
67	4	2007.11	48	M	2
68	4	2007.11	38	M	3
69	2	2007.11	54	M	1
70	2	2007.12	43	M	1
71	4	2007.12	49	M	1
72	3	2007.12	35	F	1
73	4	2007.12	60	M	1
74	3	2008.01	52	M	1
75	3	2008.01	26	M	1
76	4	2008.01	51	M	2
77	3	2008.01	51	F	1
78	2	2008.01	75	M	1
79	2	2008.02	60	M	1

80	2	2008.01	42	F	1
81	1	2008.01	42	M	1
82	4	2008.02	22	M	2
83	4	2008.02	55	M	1
84	2	2008.02	45	M	1
85	2	2008.02	48	M	1
86	4	2008.02	58	M	1
87	3	2008.02	30	M	2
88	2	2008.02	57	M	1
89	4	2008.02	28	M	1
90	4	2008.03	57	M	2
91	4	2008.03	59	M	1
92	4	2008.03	66	M	3
93	3	2008.03	66	M	1
94	4	2008.03	41	M	1
95	2	2008.03	46	F	1
96	2	2008.03	45	M	2
97	3	2008.03	53	M	1
98	2	2008.03	68	F	1
99	4	2008.04	71	M	2
100	2	2008.04	45	F	1
101	4	2008.04	36	M	3
102	4	2008.04	55	F	3
103	3	2008.04	36	M	1
104	3	2008.04	23	M	1
105	3	2008.05	60	M	1
106	2	2008.05	46	F	1
107	2	2008.05	43	M	1
108	3	2008.05	52	M	1
109	2	2008.05	36	F	1
110	2	2008.05	67	M	1
111	4	2008.05	57	M	1
112	3	2008.06	35	M	1
113	4	2008.06	48	M	1
114	2	2008.06	45	F	1
115	3	2008.06	43	F	1
116	2	2008.06	61	M	1
117	2	2008.06	54	M	1
118	3	2008.06	51	M	1
119	4	2008.06	34	F	1
120	2	2008.06	65	M	1
121	4	2008.05	57	M	1
122	3	2008.06	77	M	1

123	4	2008.06	50	M	1
124	2	2008.06	52	M	2
125	4	2008.06	60	M	1
126	3	2008.06	56	M	2
127	1	2008.06	52	M	1
128	1	2008.06	60	M	1
129	4	2008.06	36	M	1
130	2	2008.07	52	M	2
131	3	2008.07	38	F	1
132	2	2008.08	76	M	1
133	1	2008.08	52	M	1
134	4	2008.07	58	M	2
135	3	2008.07	38	F	1
136	3	2008.08	62	M	2
137	2	2008.08	43	M	1
138	2	2008.08	38	F	1
139	2	2008.08	55	F	1
140	3	2008.08	38	M	1
141	2	2008.08	38	F	1
142	4	2008.08	66	M	1
143	2	2008.09	30	F	1
144	4	2008.09	61	M	3
145	3	2008.10	35	F	1
146	2	2008.10	42	M	1
147	4	2008.10	41	F	1
148	3	2008.10	41	M	1
149	1	2008.10	26	M	1
150	4	2008.10	23	F	1

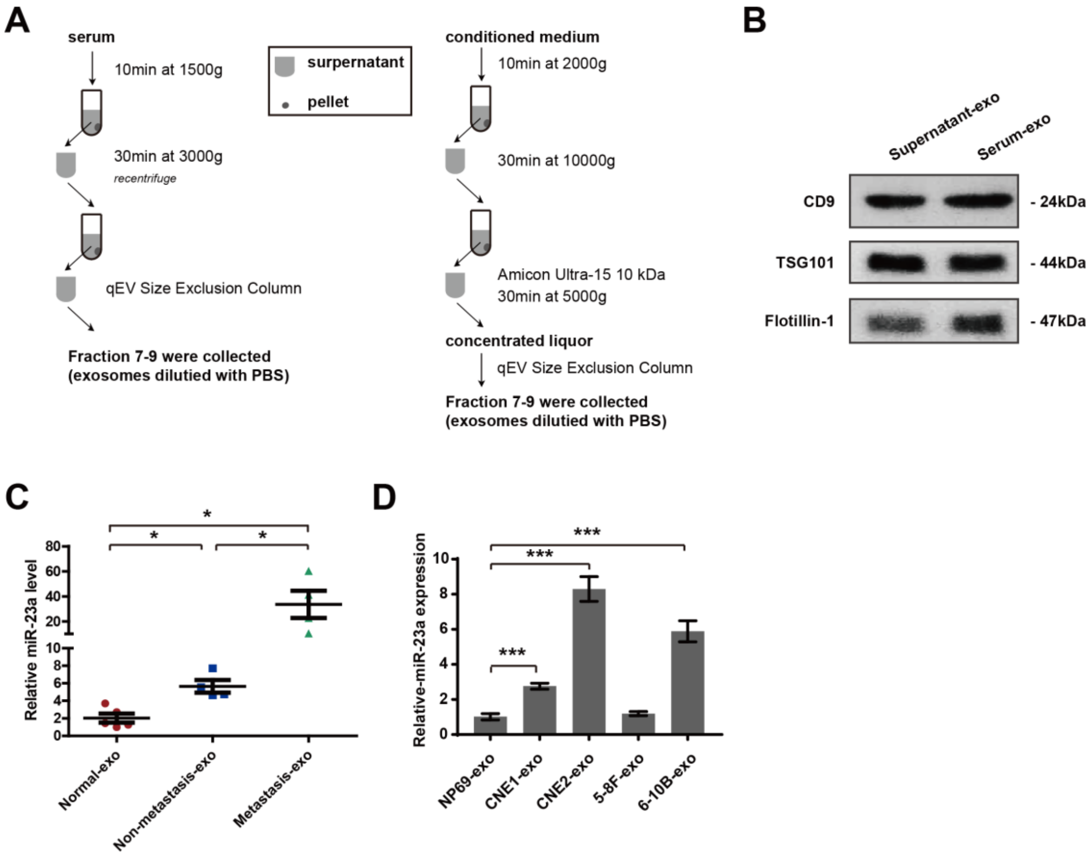

Supplymentary Figure S1: MiR-23a is highly expressed in NPC-exo by qEV Size Exclusion Columns. (a) Scheme exosomes isolation by qEV. (b) Western blot analysis of exosomal markers. Flotillin-1 was used as a loading control. (c-d) qRT-PCR of miRNA level in NPC-exosomes. Student's t-test.

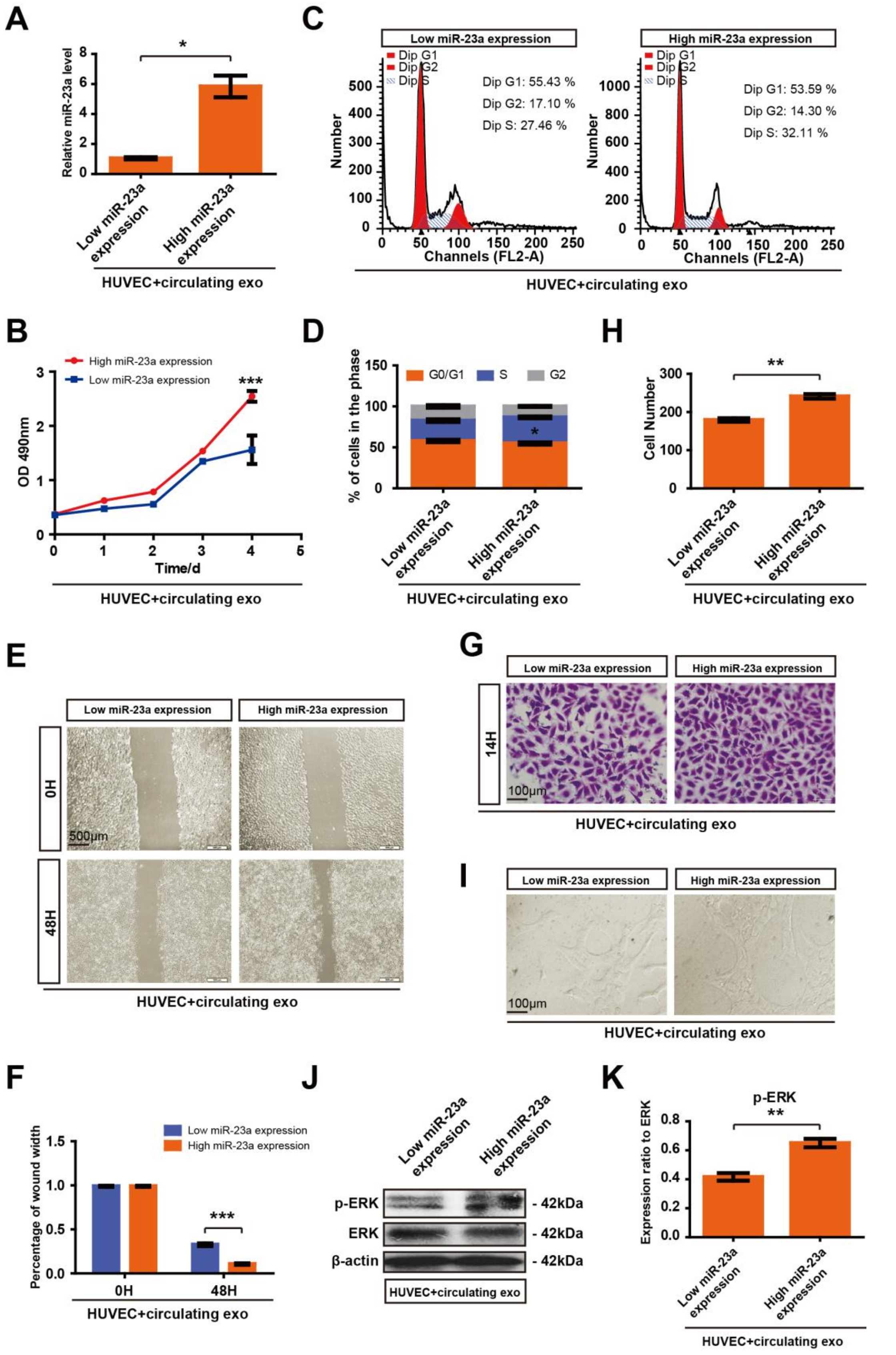

Supplymentary Figure S2: Circulating exosomes contain miR-23a modulates *in vitro* angiogenesis.

(a) 48h after treated with circulating exosomes, miR-23a levels of HUVECs were measured by

qRT-PCR. t test. (b) Viabilities of HUVECs treated with circulating exosomes were measured by the CCK8 assay. Two-way ANOVA. (c-d) Flow cytometry analysis of the cell cycle were performed 48h after treated with circulating exosomes. t test. (e-f) Wound healing assay showed migration of HUVECs treated with various exosomes. Two-way ANOVA. (g-h) Transwell migration assays were performed to measure cell migration. t test. (i) Tube formation assays using HUVECs supplemented with exosomes were conducted using Matrigel. (j-k) Western blot of p-ERK in HUVECs incubation with circulating exosomes. t test.

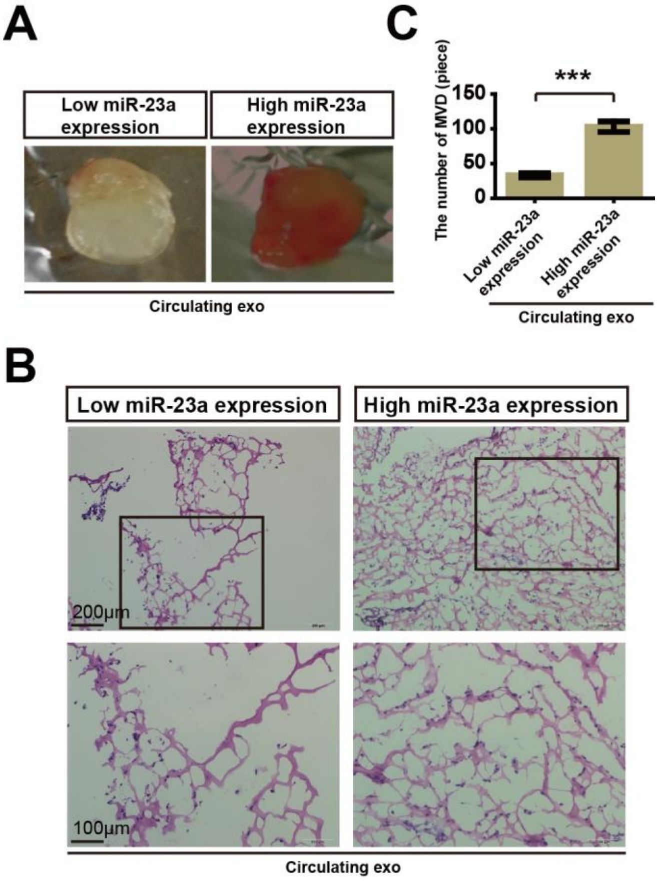

Supplymentary Figure S3: Circulating exosomes contain miR-23a modulates *in vivo* angiogenesis. (a) Gross observation, circulating exosomes modulated angiogenesis. (b) Representative micrographs of hematoxylin and eosin staining of Matrigel. (c) Quantitative evaluation of angiogenesis. Student's t-test.

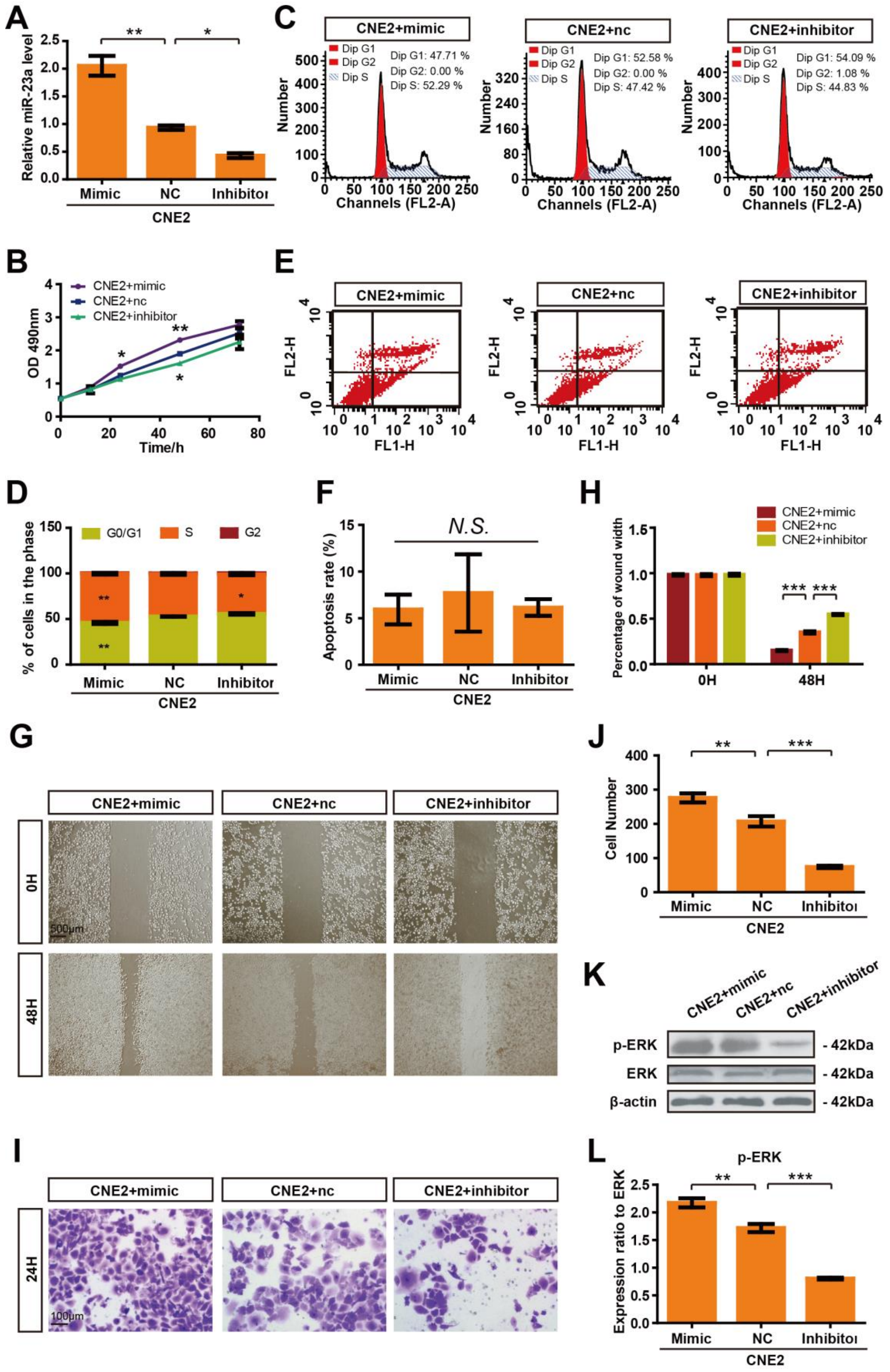

Supplymentary Figure S4: MiR-23a enhanced CNE2 cell proliferation and migration. (a) Transfection efficiency were measured by qRT-PCR. One-way ANOVA. (b) The cell growth of

transfected CNE2 cells was measured by CCK8 assay. Two-way ANOVA. (c-d) Flow cytometry analysis of the cell cycle were performed at 36 after transfection. One-way ANOVA. (e-f) Apoptosis rates were measured by FCM analysis after Annexin V/PI staining. One-way ANOVA. (g-h) Wound healing assay showed cell migration in transfected CNE2 cells. Two-way ANOVA. (i-j) Transwell migration assays were performed to measure cell migration. One-way ANOVA. (k-l) images of p-ERK expression in transfected cells. One-way ANOVA.

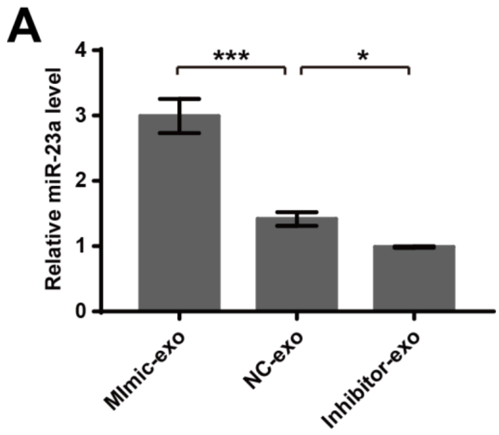

Supplymentary Figure S5: Exosomal miR-23a expression predominantly changed in miR-23a-treated cells by qEV Size Exclusion Columns. (a) qRT-PCR of miRNA expression in exosomes isolated from HUVECs treated as indicated. One-way ANOVA.

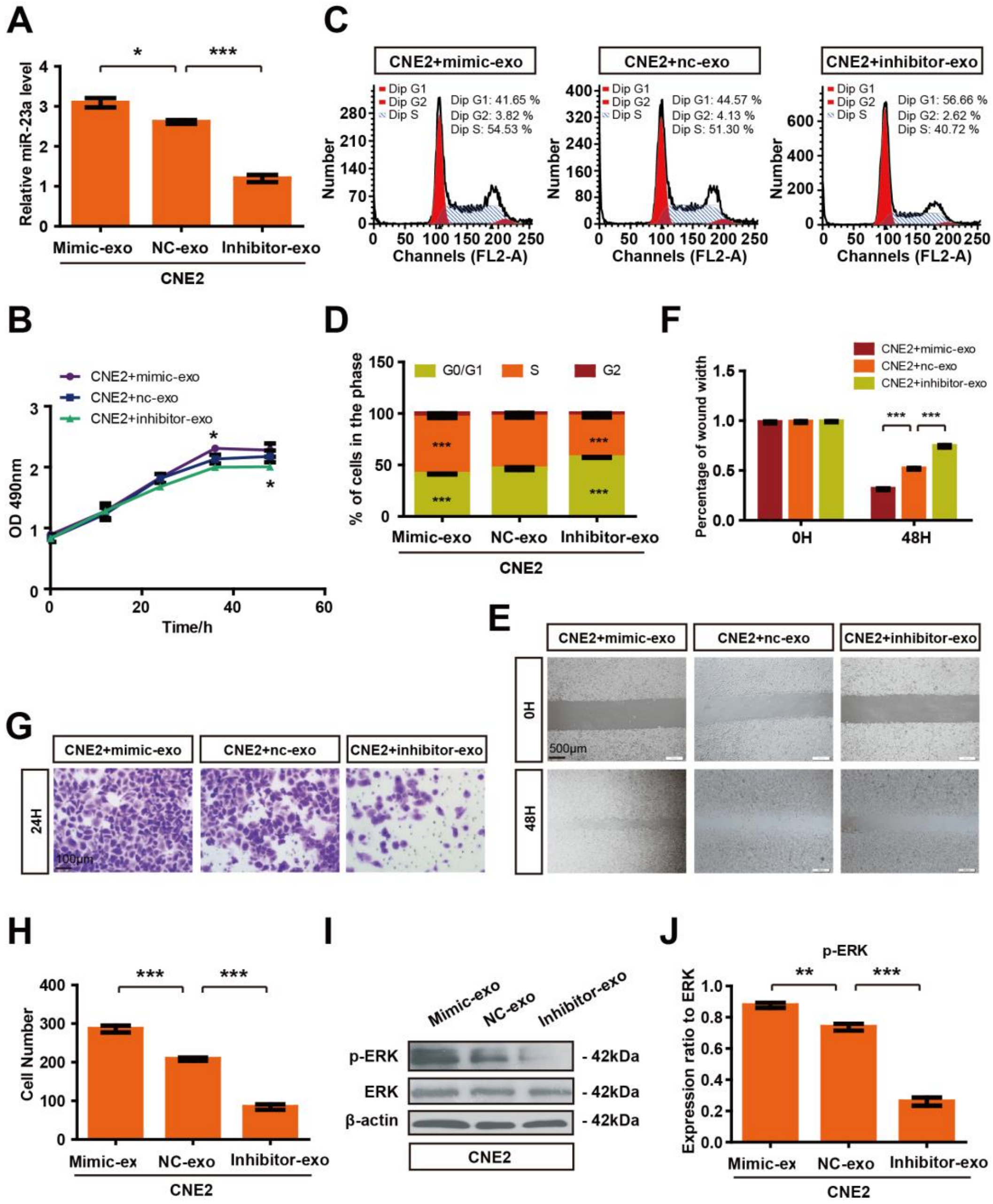

Supplymentary Figure S6: High exosomal miR-23a promoted CNE2 cell proliferation and migration. (a) 48h after treated with exosomes isolated from culture medium of transfected CNE-2 cells, miR-23a levels of CNE2 were measured by qRT-PCR. One-way ANOVA. (b) Viabilities of CNE2 cells treated with various exosomes were measured by the CCK8 assay. Two-way ANOVA. (c-d) Flow cytometry analysis of the cell cycle were performed 48h after treated with exosomes. One-way ANOVA. (e-f) Wound healing assay showed migration of CNE2 cells treated with various exosomes. Two-way ANOVA. (g-h) Transwell migration assays were performed to measure cell migration. One-way ANOVA. (i-j) Western blot of p-ERK in CNE2 cells incubation with exosomes. One-way ANOVA.

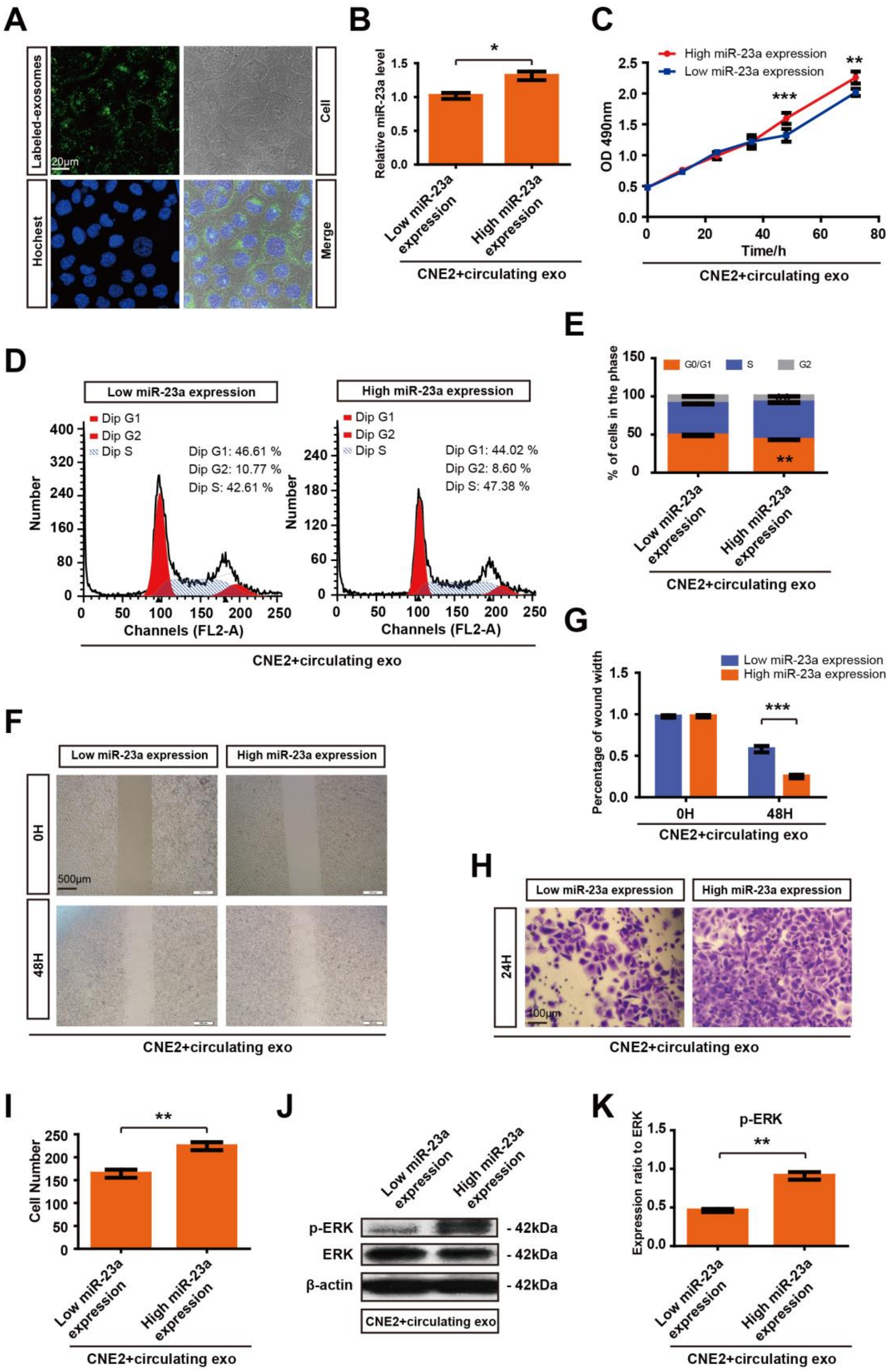

Supplymentary Figure S7: Circulating exosomes contain miR-23a modulates proliferation and migration of NPC cells. (a) Uptake of exosomes in CNE2 cells by confocal microscopy. Blue: Hoechst staining; Green: PKH67-labeled exosomes. (b) 48h after treated with circulating exosomes,

miR-23a levels of CNE2 cells were measured by qRT-PCR. t test. (c) Viabilities of CNE2 cells treated with circulating exosomes were measured by the CCK8 assay. Two-way ANOVA. (d-e) Flow cytometry analysis of the cell cycle were performed 48h after treated with circulating exosomes. t test. (f-g) Wound healing assay showed migration of CNE2 cells treated with various exosomes. Two-way ANOVA. (h-i) Transwell migration assays were performed to measure cell migration. t test. (j-k) Western blot of p-ERK in CNE2 cells incubation with circulating exosomes. t test.

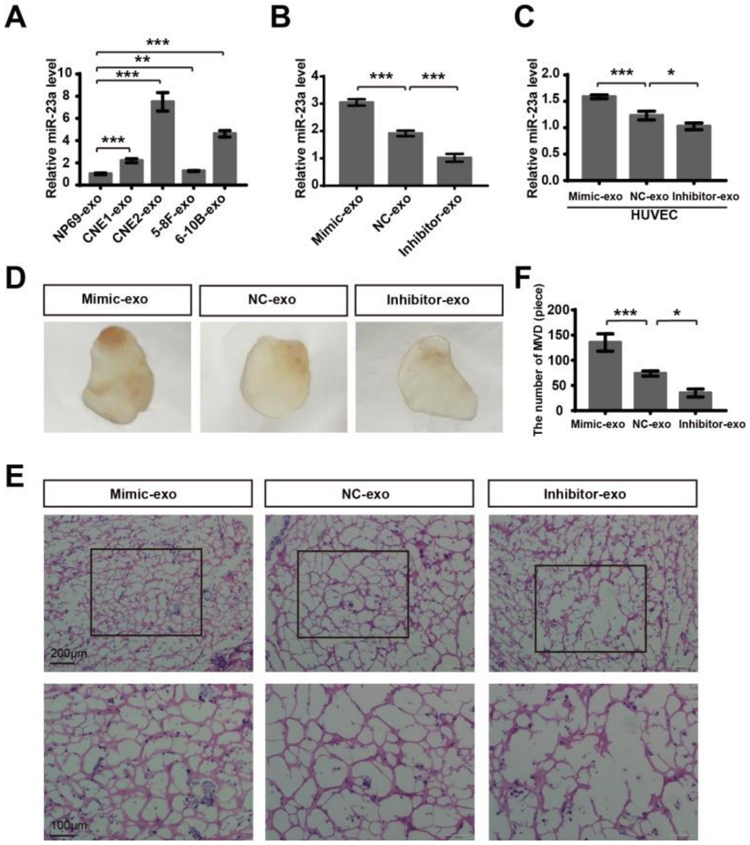

Supplymentary Figure S8: High exosomal miR-23a promoted angiogenesis using exosomes isolated from cellular media supplemented with exosome-free serum (SBI SystemBiosciences). (a) qRT-PCR of miRNA level in exosomes isolated from NPC cells. t-test. (b) qRT-PCR of miRNA expression in exosomes isolated from miR-23a-treated-CM. One-way ANOVA. (c) 48h after treated with exosomes isolated from culture medium of transfected CNE-2 cells., miR-23a levels of HUVECs were measured by qRT-PCR. One-way ANOVA. (d) Gross observation, exosomes modulated angiogenesis. (e) Representative micrographs of hematoxylin and eosin staining of Matrigel. (f) Quantitative evaluation of angiogenesis. One-way ANOVA.
